# Investigation of In Vitro Drug Release from Porous Hollow Silica Nanospheres Prepared of ZnS@SiO_**2**_ Core-Shell

**DOI:** 10.1155/2013/541030

**Published:** 2013-09-19

**Authors:** Leila Vafayi, Soodabe Gharibe

**Affiliations:** Department of Science, Firoozkooh Branch, Islamic Azad University, Firoozkooh 148, Iran

## Abstract

In this contribution, porous hollow silica nanoparticles using inorganic nanosized ZnS as a template were prepared. The hydrothermal method was used to synthesize pure ZnS nanospheres material. The ZnS@SiO_2_ core-shell nanocomposites were prepared using a simple sol-gel method successfully. The hollow silica nanostructures were achieved by selective removal of the ZnS core. The morphology, structure, and composition of the product were determined using powder X-ray diffraction (XRD), emission scanning electron microscopy (SEM), transmission electron microscopy (TEM), and Fourier transform infrared spectroscopy (FT-IR). The results demonstrated clearly that the pure ZnS nanoparticles are in a spherical form with the average size of 40 nm and correspond with zinc blend structure. The porous hollow silica nanoparticles obtained were exploited as drug carriers to investigate in vitro release behavior of amoxicillin in simulated body fluid (SBF). UV-visible spectrometry was carried out to determine the amount of amoxicillin entrapped in the carrier. Amoxicillin release profile from porous hollow silica nanoparticles followed a three-stage pattern and indicated a delayed release effect.

## 1. Introduction

During the past decade, there have been widespread research efforts to develop architecture and fabrication of core-shell composite materials and hollow spheres of nanometer to micrometer size with special physical and chemical properties. They have demanding applications in pharmaceuticals, biology, optics, catalysts, and drug delivery [1–7]. 

II-VI semiconductor nanocrystals attract much attention because of their size dependent photo and electroluminescence properties and promising applications in optoelectronics [8–11]. Among the family of II-VI semiconductors, zinc sulfide semiconductor is an important member of this family because of its favorable electronic and optical properties for optoelectronic applications. ZnS can have two different crystal structures (zinc blende and wurtzite); both have the same band gap at 340 nm (3.66 eV) and the direct band structure. ZnS has been used widely as an important phosphor for photoluminescence (PL), electroluminescence (EL), and cathodoluminescence (CL) devices, due to its better chemical stability compared to other chalcogenides, such as ZnS. In optoelectronics, it finds use as light emitting diode, reflector, dielectric filter, and window material [12–15]. Due to the fact that SiO_2_ coating semiconductor nanocrystals have an interesting field of study, they are used as bioconjugation and excellent luminescent probes [16, 17].

The controlled release of drugs from an inert matrix has become increasingly important for oral, implantable, and transferal therapeutic systems, due to the advantages of safety, effectiveness, and patient accessibility [18–21]. Several research groups have studied the drug adsorption and release properties of mesoporous silica materials. Among a variety of nanoparticle-based drug delivery systems, mesoporous silica nanoparticles have several advantageous features for use in the delivery of both water soluble and insoluble drugs. These materials have large surface areas and porous interiors that can be used as reservoirs for storing the drug. The pore size and environment can also be modified to selectively store different molecules, while the size and shape of the nanoparticles can be tuned to enhance cellular uptake process. Furthermore, robust inorganic materials do not swell in organic solvents and are stable at varying pH conditions. An ideal mesoporous carrier with a high specific surface area, large pore volume, and appropriate pore size (larger than the kinetic diameter of the drug) would be beneficial toward increasing the adsorption capacity [22–27].

Inorganic hollow particles are more remarkable due to their excellent properties such as low density, specific surface area, thermal insulation, and permeability [28]. Several methods have been used to prepare hollow particles such as a polymer bead template method [29], an inorganic template method [30], a sol-gel method used for surfactant stabilized emulsions [31], spray drying [32], a spray precipitation method [33], and spray pyrolysis [34]. Recently, bacterial cells have been used as a template to fabricate hollow structures [35, 36].

In this work, we reported a method for preparation of hollow silica nanospheres with porous shell structure by the sol-gel technique, using inorganic ZnS nanoparticles as a template. The template of ZnS nanospheres was prepared by the hydrothermal method. Subsequently, the surfaces of ZnS nanospheres were coated with SiO_2_ by a simple and low cost method. The hollow silica nanospheres were obtained via the selective removal of ZnS cores. At last, the porous hollow silica nanoparticles obtained were exploited as drug carriers to investigate in vitro release behavior of amoxicillin in simulated body fluid.

## 2. Experimental

All reagents were of analytical grade and used without any further purification.

In order to obtain a pure phase of monodispersed ZnS nanospherical particles as a template for hollow SiO_2_ nanostructures, the synthesized conditions such as the effects of different sources of Zn and S, type of the surfactant, the reaction temperature, and time have been optimized. In this work, we report only the conditions which in a pure phase of monodispersed ZnS nanospherical particles have been obtained.

### 2.1. Synthesis of ZnS Nanosphere Particles

 A solution of 3 mmol ZnCl_2_ in 20 mL deionized distilled water was added to a solution of surfactants with ZnCl_2_ to CTAB ratio equal to 1 and prepared in 20 mL of deionized water under stirring. Then, 3 mmol of Na_2_S in 20 mL deionized distilled water was added to the above mixture under vigorous stirring. The mixture was transferred into an autoclave, sealed, and kept at 120°C for 5 h. After cooling the system to room temperature, the product was separated by centrifugation, washed with absolute ethanol and deionized water for several times, and then dried under vacuum at 70°C for 10 h.

### 2.2. Synthesis of ZnS@SiO_**2**_ Core-Shell Nanocomposites and Hollow Silica Nanostructures

In a typical synthesis, about 0.1 g of ZnS particles and 0.06 g of CTAB were added to 50 mL of absolute ethanol and sonicated for 20 min using an ultrasound cleaner. Afterwards, 10 mL NH_4_OH and 50 *μ*L tetraethyl orthosilicate (TEOS) (molar ratio of ZnS : TEOS : CTAB equal to 6 : 2 : 1) were added to the mixture and sonicated for 4 h. Then, the product was separated by centrifugation, washed with absolute ethanol and deionized water for several times, and at last dried under vacuum at 60°C for 4 h.

In order to reach ZnS and obtain hollow silica shell, the ZnS/SiO_2_ core-shell nanocomposites were added into HNO_3_ (1 M) and kept for two weeks. The resulting product was separated by centrifugation, washed with deionized water for several times, and then dried under vacuum at 60°C for 6 h.

### 2.3. Drug Loading and Release

The loading of the drug was accomplished by the soaking of hollow silica samples in amoxicillin aqueous solution with a certain concentration. 

In a typical loading procedure, hollow silica powders were soaked in 50 mL solution of 120 ppm of amoxicillin (weight ratio of amoxicillin : SiO_2_ = 2 : 1). The suspension was stirred vigorously for 48 h. The drug-loaded nanoparticles were centrifuged and washed with acetone three times and then dried under vacuum at 40°C for 3 h. The amount of drug loaded on the nanoparticles was investigated measuring the adsorption intensity of the amoxicillin remaining in the solution by means of UV-Vis spectroscopy (Shimadzu-2550). 

In vitro release of amoxicillin was performed by soaking the drug-loaded nanoparticles powder in simulated body fluid (SBF), with continuous stirring at 37°C in a thermostatic water bath. The release ratio of amoxicillin from the drug-loaded nanoparticles was measured by examining the concentrations of amoxicillin in SBF at different time intervals by means of UV-Vis spectroscopy. 

### 2.4. Characterization

The crystal phase and particle size of the synthesized products were characterized by X-ray diffraction (XRD) using FK60-04 with Cu K*α* radiation (*λ* = 1.54 Å) and with instrumental settings of 35 kV and 20 mA. The morphology of the nanostructures was observed by emission scanning electron microscopy (SEM, Philips-XL*φ*30) and transmission electron microscopy (TEM, Philips-CM120). Fourier transform infrared (FT-IR) spectra were recorded on a Shimadzu-840S spectrophotometer using KBr pellet.

## 3. Results and Discussion

**Figure 1:**
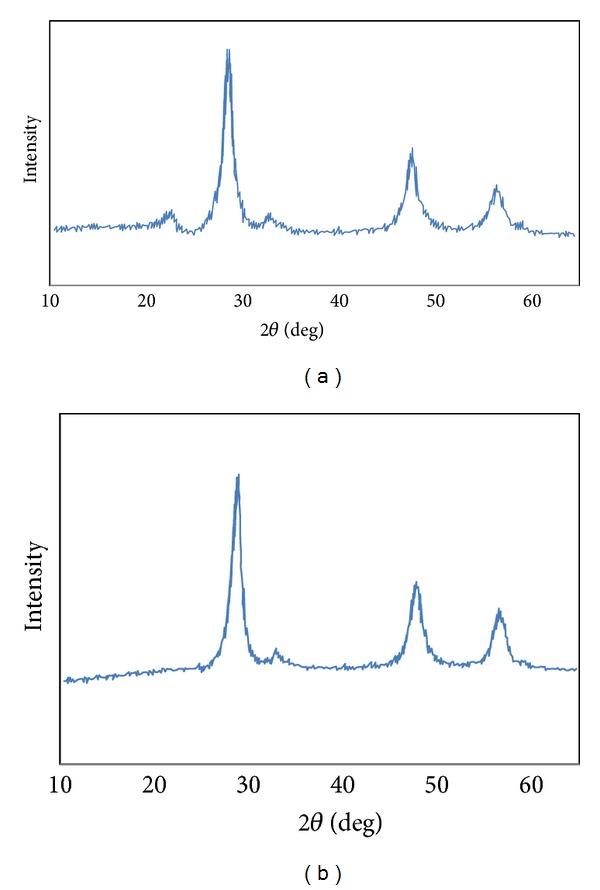
XRD patterns: (a) ZnS@SiO_2_ core-shell nanocomposites and (b) bare ZnS nanoparticles.

The XRD patterns of pure ZnS nanoparticles (b) and ZnS@SiO_2_ core-shell nanocomposites (a) are shown in Figure 1. All peaks can be well indexed to cubic zinc blend (JCPDS, Card No. 05-0566, *a* = *b* = *c* = 5.406 (8) Å). No other crystalline phase was found in the XRD pattern. The lattice constant obtained from XRD data was *a* = 5.405 Å. This result indicated that the lattice parameter of the nanoparticles is smaller than those of the bulk crystalline ZnS (*a* = 5.406 Å). The result also shows that in most reported nanoparticles, the lattice constant often decreases with decreasing the particle size [7, 37, 38]. In Figure 1(a) a broad new peak appeared at diffraction degree about 22°, which presents the SiO_2_ amorphous state in the product. Also, the peaks of ZnS@SiO_2_ core-shell nanocomposites (Figure 1(a)) are a little broader with less intensity than those of the pure ZnS nanoparticles (Figure 1(b)), which is probably due to the presence of SiO_2_ in an amorphous state around the ZnS nanoparticles.

**Figure 2:**
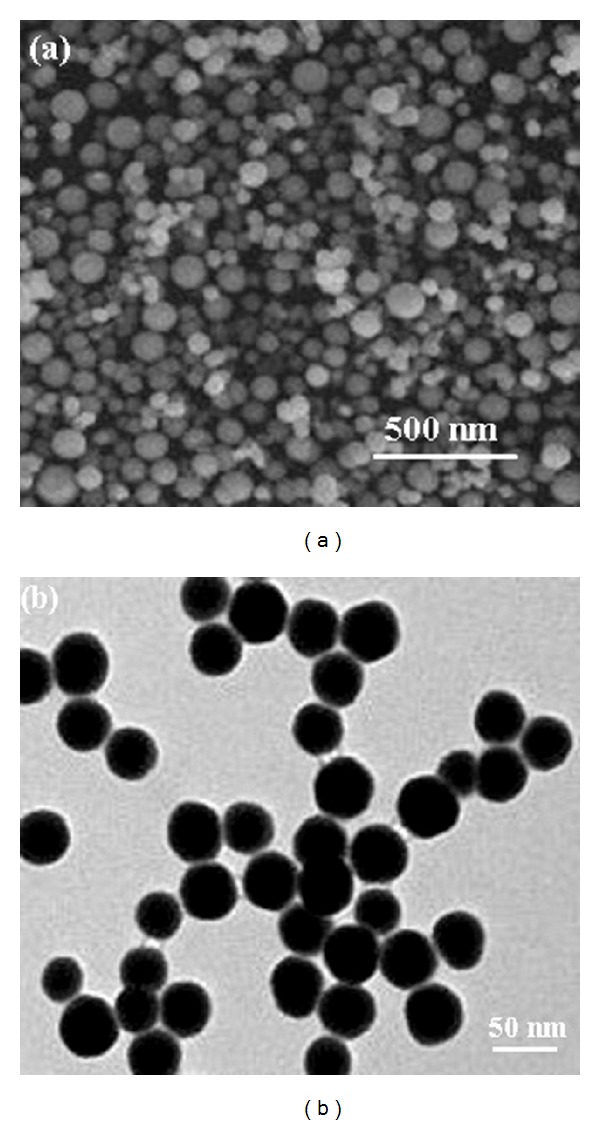
(a) SEM and (b) TEM images of ZnS nanospheres.

**Figure 3:**
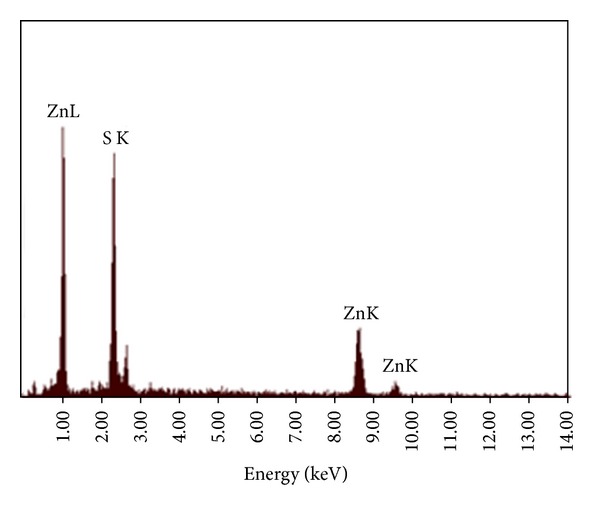
EDX spectrum of ZnS nanospheres.

**Figure 4:**
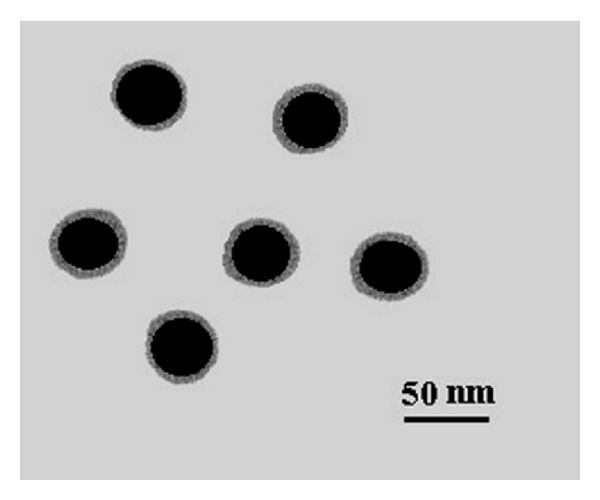
TEM image of ZnS@SiO_2_ core-shell nanocomposites.

**Figure 5:**
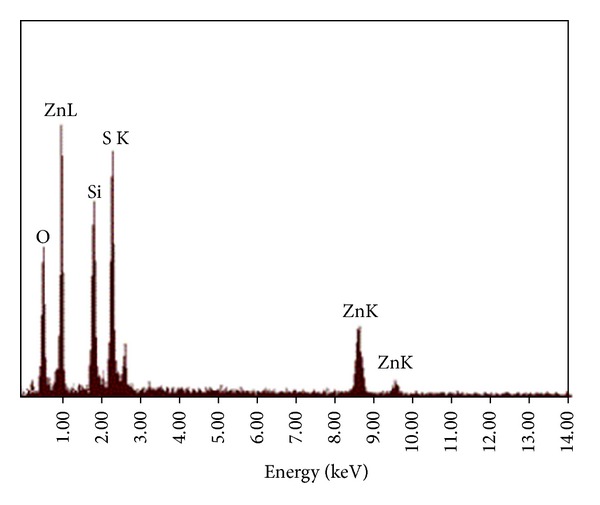
EDX spectrum of ZnS@SiO_2_ core-shell nanocomposites.

Figure 2 shows the SEM and TEM images of the ZnS nanoparticles. These images clearly demonstrate that the products are spherical, with average diameter of 40 nm. The energy dispersive X-ray (EDX) analysis of prepared sample confirms that the product consists of Zn, S, and the Zn and S elemental ratio is 1 : 1 (Figure 3). Also, the SiO_2_ shell around the ZnS core is clearly obvious in the TEM image of ZnS@SiO_2_ core-shell nanocomposites (Figure 4). It is clear that ZnS@SiO_2_ core-shell nanocomposites are larger than the ZnS nanoparticles without the shell. The thickness of the SiO_2_ shell is about 10 nm, which can be controlled by changing the amount of ZnS to TEOS ratio [16, 17]. The elemental analysis of ZnS@SiO_2_ core-shell nanocomposites confirms the presence of Si in the product (Figure 5).

**Figure 6:**
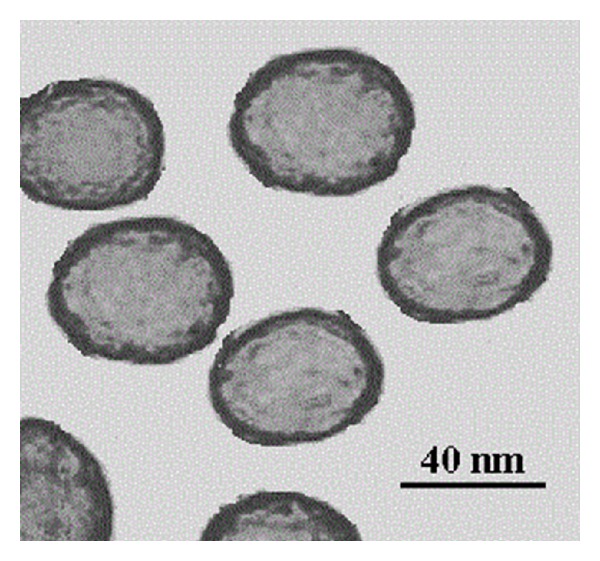
TEM image of hollow silica nanostructures.

TEM image at Figure 6 shows the hollow silica shells. The size of the spherical silica nanoparticles is around 40 nm in diameter with wall thickness of about 10 nm. The shape and size of the hollow silica can be controlled by employing different nanosized ZnS templates. The SiO_2_ shells are obtained by removing the ZnS templates from ZnS@SiO_2_ core-shell particles by etching in HNO_3_ aqueous solution. 

**Figure 7:**
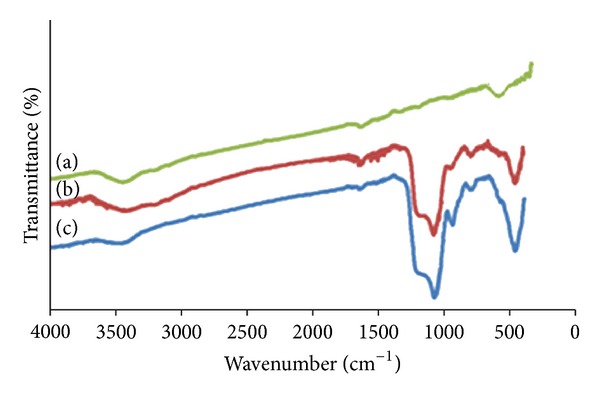
FT-IR spectra of (a) bare ZnS nanoparticles, (b) ZnS@SiO_2_ core-shell nanocomposites, and (c) hollow SiO_2_ nanostructures.

The FT-IR spectra of pure ZnS nanoparticles, ZnS@SiO_2_ core-shell nanocomposites, and hollow silica nanostructures are shown in Figures 7(a)–7(c), respectively. The peak at 600–620 cm^−1^ is assigned to ZnS band (i.e., corresponding to sulphides) [10]. The vibrational peaks in the range of 3350–3450 cm^−1^ and 1600–1650 cm^−1^ can be attributed to the stretching and bending vibrations of structural hydroxyl groups of the adsorbed water [4, 16]. The peaks in the ranges of 1050–1110 cm^−1^ and 750–800 cm^−1^ in Figures 7(b) and 7(c) correspond to the asymmetric and symmetric stretching vibration modes of the Si–O–Si, respectively, and are absent in Figure 7(a). Also, the peak which appears at 440–480 cm^−1^ is due to the bending vibration mode of Si–O–Si [4, 16]. These results clearly show that silica is successfully coating the ZnS nanoparticles.

**Figure 8:**
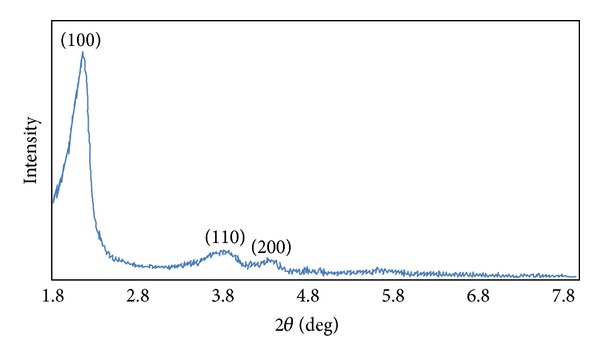
Low angle XRD pattern of silica nanoshells.

Figure 8 shows a low angle XRD pattern of silica nanoshells. It clearly indicates three peaks indexed as (100), (110), and (200), which can be associated with well-ordered 2D hexagonal mesostructure [39].

Semiconductor nanocrystals with SiO_2_ coating are used as bioconjugation and excellent luminescent probes. Also, the hollow nanostructures via selective removal of ZnS attract the attention of many researchers in applying them in drug delivery and catalysis.

In this work, the hollow silica nanospheres obtained were employed for drug delivery application. The results of FT-IR and TEM exhibited the formation of hollow spherical SiO_2_. The specific surface area (BET) of the obtained shells is 895 m^2^/g. After the loading of the drug, the specific surface area has been obtained to be 362 m^2^/g. The decrease of the specific surface area of the nanoshells is due to the loading of the drug in the inner core and pores of the nanoshells. This confirms that amoxicillin was adsorbed not only on external surfaces of the silica nanoparticles, but also mainly inside the pores. SiO_2_-drug conjugation methods include silanization and electrostatic attraction. Amoxicillin contains amino and hydroxyl groups, which can interact with silanol groups in silica via hydrogen bonding.

**Figure 9:**
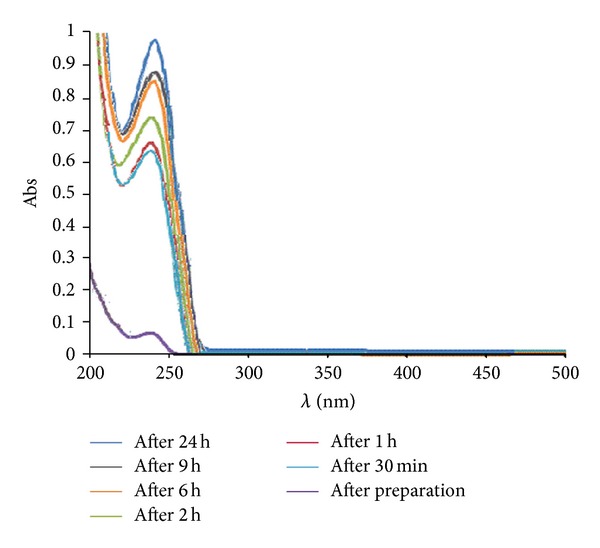
UV-Vis spectra of release of amoxicillin in SBF at the beginning and 30 min, 1 h, 2 h, 6 h, 9 h, and 24 h after the preparation of the suspension.

The drug release in vitro has been investigated by means of UV-Vis spectroscopy. The spectra were taken at the beginning and 30 min, 1 h, 2 h, 6 h, 9 h, and 24 h after the preparation of the suspension (Figure 9). As seen in Figure 7, it can be found that with the passage of time, the intensity of the adsorption peaks increases. The increase of the intensity of the adsorption peaks is due to the increase of concentration of amoxicillin in the simulated body fluid (SBF).

**Figure 10:**
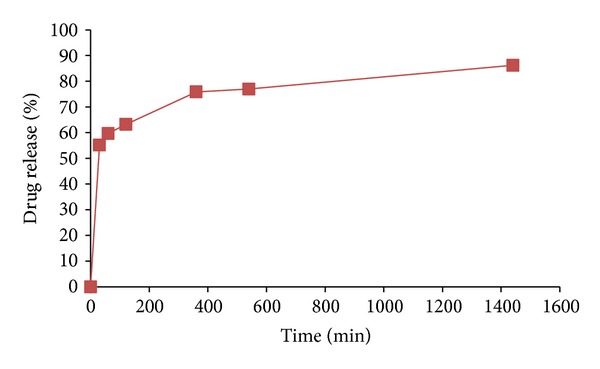
In vitro release profile of amoxicillin from porous hollow silica nanospheres (std. deviation = 21.47).

Figure 10 shows the release profile of amoxicillin from the hollow carrier. The drug release has happened in three stages. At the first stage, 55.2% of the drug has been discharged within 30 min, which is probably due to the rapid release of the drug loosely adsorbed on the surface of the nanoshells or free amoxicillin molecules. However, this type of fast release is not favorable for the practical controlled release of drug. The second stage has lasted about five and half hours, in which 20.6% of the drug was released from the pores of the nanoshells, and in the third stage, which has lasted for 18 h, 9.2% of the drug was released. It is related to the release of located drug inside the hole of nanoshells. Also, it is probable that slow release is pertained to the strong chemical adsorption of amoxicillin molecules in the silica. The remaining 15% which is maintained inside the hole of nanoshells needs much more time to release. It can be concluded that the porous hollow silica nanospheres carrier markedly delayed the release of amoxicillin and can be employed in drug delivery application.

## 4. Conclusions

A novel template for preparation of hollow silica nanoparticles using ZnS nanospheres was developed. ZnS@SiO_2_ core-shell nanocomposites were successfully synthesized by a simple chemical method. The hollow nanostructures were obtained with a diameter of 40 nm and wall thickness of approximately 10 nm via selective removal of ZnS from ZnS@SiO_2_ core-shell nanocomposites. The ZnS@SiO_2_ core-shell nanocomposites and hollow SiO_2_ nanostructures are highly favorable for nanoelectronic devices, drug delivery, and catalyst. The results showed that the porous hollow silica nanoparticles obtained were exploited as drug carriers to investigate in vitro release behavior of amoxicillin in simulated body fluid. Amoxicillin release profile from porous hollow silica nanoparticles followed a three stage pattern, which was explained as the drug release from surface, pore channels in the wall, and the inside hollow part of the hollow silica nanospheres and indicated a delayed release effect. Therefore, the porous hollow silica nanospheres can be employed in drug delivery application.
